# Understanding healthcare practices in superdiverse neighbourhoods and developing the concept of welfare bricolage: Protocol of a cross-national mixed-methods study

**DOI:** 10.1186/s12914-015-0055-x

**Published:** 2015-06-28

**Authors:** Jenny Phillimore, Hannah Bradby, Michi Knecht, Beatriz Padilla, Tilman Brand, Sin Yi Cheung, Simon Pemberton, Hajo Zeeb

**Affiliations:** Institute for Research into Superdiversity (IRiS), School of Social Policy, University of Birmingham, Edgbaston, Birmingham, B15 2TT Great Britain; Department of Sociology, Uppsala University, Box 624, Se-751 26 Uppsala, Sweden; Department of Anthropology and Cultural Research, University of Bremen, Enrique-Schmidt-Straße 7, 28359 Bremen, Germany; Interdisciplinary Centre of Social Sciences (CICS.NOVA), University of Minho, Campus de Gualtar, 4710-057 Braga, Portugal; Leibniz Institute for Prevention Research and Epidemiology - BIPS GmbH, Achterstraße 30, D-28359 Bremen, Germany; School of Social Sciences, Cardiff University, King Edward VII Avenue, Cardiff, CF10 3W Great Britain; Keele University, Keele, Staffordshire, ST5 5UK Great Britain; Health Sciences Bremen, University of Bremen, Bremen, Germany

**Keywords:** Access to healthcare, Health inequality, Superdiversity, Welfare state, Bricolage

## Abstract

**Background:**

Diversity in Europe has both increased and become more complex posing challenges to both national and local welfare state regimes. Evidence indicates specific barriers for migrant, faith and minority ethnic groups when accessing healthcare. However, previous studies of health in diverse cities in European countries have mainly adopted an ethno-national focus. Taking into account the new complexity of diversity within cities, a deeper and multi-faceted understanding of everyday health practices in superdiverse contexts is needed to support appropriate healthcare provision.

**Methods/Design:**

This protocol describes a mixed method study investigating how residents in superdiverse neighbourhoods access healthcare. The study will include participant observation and qualitative interviewing as well as a standardised health survey and will be carried out in eight superdiverse neighbourhoods – with varying deprivations levels and trajectories of change – in four European countries (Germany, Portugal, Sweden and UK). In each neighbourhood, trained polylingual community researchers together with university researchers will map formal and informal provision and infrastructures supportive to health and healthcare. In-depth interviews with residents and healthcare providers in each country will investigate local health-supportive practices. Thematic analysis will be used to identify different types of help-seeking behaviours and support structures across neighbourhoods and countries. Using categories identified from analyses of interview material, a health survey will be set up investigating determinants of access to healthcare. Complex models, such as structural equation modelling, will be applied to analyse commonalities and differences between population groups, neighbourhoods and countries.

**Discussion:**

This study offers the potential to contribute to a deeper understanding of how residents in superdiverse neighbourhoods deal with health and healthcare in everyday practices. The findings will inform governmental authorities, formal and informal healthcare providers how to further refine health services and how to achieve equitable access in diverse population groups.

## Background

Unequal access to healthcare has been recognized as one major reason for health inequities [1–4]. Migrants and ethnic minorities have been shown to experience problems when accessing healthcare. Although migrants do not show consistent under- or over-use of services compared to the general population, systematic reviews indicate distinct patterns of healthcare utilisation among migrants in Europe [5, 6]. However, previous studies on migrant health in European countries have mainly focused on well-established minorities in single country-settings such as migrants from South Asia and Caribbean regions in the United Kingdom (UK) or the so-called guest workers from Turkey and the ethnic Germans (‘Aussiedler’) from the former Soviet Union in Germany. This ethno-national focus does not adequately take into account increasing and more complex diversity among new migrant, minority and resident populations. Superdiversity is the process of inter- and intra-group diversification triggered by increased globalised mobility [7], resulting in a level of population complexity and heterogeneity, and a faster pace of change than ever previously witnessed [8, 9]. Superdiversity comprises a quantitative dimension in terms of the increase in arrival of migrants from a wider range of ethnicities and/or countries of origin and a qualitative dimension focusing on intra- and inter-group diversity. The “diversification of diversity” [10] recognises that groups previously considered homogenous are increasingly differentiated by immigrant statuses, welfare entitlements, types of participation in the labour market, gender and age profiles, and local as well as translocal or transnational support structures. Responses to such diversification by health, social or other services require a more nuanced understanding of the lived complexities of those residing in superdiverse neighbourhoods, and the importance of fluidity, hybridity and relationality. Examining the interplay of such factors aids the avoidance of reifying ethnicity as an essentialised cause of poor health [11, 12]. Superdiverse neighbourhoods are so-called “arrival zones” [13] usually receiving large numbers of new migrants, and established minorities alongside an often impoverished and/or elderly less-mobile majority group [14, 15], however these territories may also be subject to gentrification or other upward mobility trends related to policies that promote urban renewal and cosmopolitan conviviality.

Inequalities in health and access to healthcare vary across European countries. National differences in outcomes have sometimes been linked to welfare state regimes [16–20]. While welfare state theory had been dominated by welfare policy analysis with debates around fitting countries to typologies [21, 22], local welfare systems have received increasing attention. The rationale for localising welfare systems responds to the argument that as societies become more complex, individual needs are best met through empowering residents to work with local actors to tailor services to meet individual needs [23]. Further the emergence of welfare chauvinism as a tool of immigration control has the potential to exclude some residents, particularly in superdiverse areas which have higher concentrations of non-citizens, whose access to services is shaped by immigration regimes linked to welfare exclusions [24]. The challenge for local systems is to provide good quality, accessible health services and preventive services, without reverting to re-familiarisation processes and thereby increasing inequality and vulnerability [23]. If we are to understand the impact of welfare states upon health, and the potential to meet the health needs of complex populations in superdiverse neighbourhoods, we must explore how access and experience vary by both national welfare state characteristics and local experience [25] as well as the different worlds of welfare chauvinism that emerge from immigration regimes [26]. The emergence of specific demographic configurations such as superdiversity, and the imperative to reduce costs, all support a shift to the local where messy, complex, unexpected forms of everyday strategies that combine, mix and link different resources, can be investigated and understood. Interviews with a maximum diversity sample in transnational focus and across four European settings shows that complexity and contingency characterise help-seeking strategies for health and that help was sought by new and long-standing residents, without a discernible pattern according to the relevant welfare regimen [27]. This implies potential for the development of a new concept to aid understanding of local actions: that of welfare bricolage.

Most of the previous research on access to healthcare has focused on formal services [28–30]. Adopting the concept of welfare bricolage shifts the focus towards studying the processes whereby residents combine informal, formal and internet provision in an attempt to meet needs. Bringing superdiversity and bricolage together alerts us to the importance of context, and the difference that space, and residents make to the practices of welfare systems. Considering the “global sense of place” [31] in superdiverse neighbourhoods, welfare bricolage can be viewed as a fluid, relational, hybrid process, defined and operationalised from (inter)connections with elsewhere as well as from within. De Certeau’s work on the “practice of everyday life” [32] leads us to explore how the tactic of bricolage may be utilized by residents accessing different forms of healthcare. Superdiversity and bricolage as concepts support a radical and empirically-informed interrogation of established wisdom about healthcare in welfare systems to produce new, locally-informed thick descriptions about healthcare practices in superdiverse populations which employ collaborative models of knowledge production to develop new models of scientific and practical relevance.

This protocol describes the study “Understanding the practice and developing the concept of welfare bricolage” (UPWEB). Using a wide definition of health as including social aspects of wellbeing [33], the UPWEB study aims to investigate the experiences and approaches of residents in superdiverse areas and examine the role of providers including the formal, informal, public, private, third sector and internet-based services. The study objectives are:

- To examine residents’ experiences of accessing and communicating with providers and the approaches residents take to optimise their access to healthcare.
- To investigate the factors which influence people’s access to, and experiences of, healthcare including local and national welfare states, health and migration regimes.
- To explore the ways in which different types of providers identify need and investigate the roles they adopt, and challenges and opportunities they face.
- To advance new methods capable of collecting data about welfare in highly complex spaces.

## Methods/Design

**Fig. 1:**
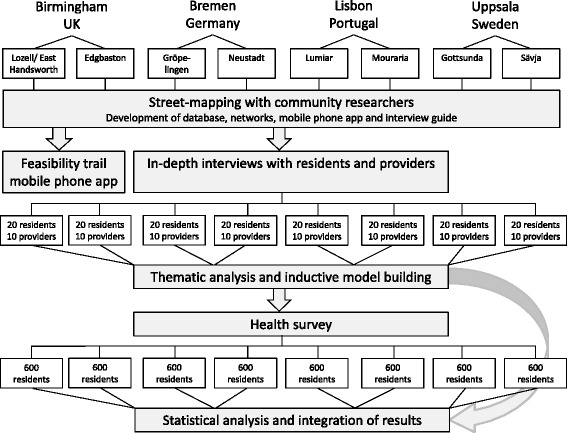
UBWEB study design

In the UPWEB study a multidisciplinary team uses a mixed methods design including ethnographic observation, interviewing and a standardised health survey. The study will be implemented in eight superdiverse neighbourhoods in four European countries (Germany, Portugal, Sweden and UK) adopting a within and between country comparative approach. Each country represents an a priori different type of welfare state regime according to established regime typologies [34]. Within the countries, two neighbourhoods in one city are selected which are both characterised by superdiversity but with one of them displaying a high degree of deprivation and the other showing upward social mobility (Table 1). The study design comprises three stages: a mapping of health-related activities and infrastructures of the neighbourhoods (street-mapping), in-depth interviews and participant observation with residents and service providers and a health survey (Fig. 1). The study began in January 2015 and continues until December 2017.

**Table 1:** Characteristics of the comparison countries and neighbourhoods

	City	Selection criteria	Neighbourhood
Germany	Bremen	Corporatist/Conservative regime.	Gröpelingen: 35,055 residents, 44.1 % PMB, 2^nd^ highest number welfare dependants (33.3 %), high deprivation. Long history migration.
	10^th^ largest city	Immigrants must prove lawful residence.	
	550,406 residents, 24.54 % PMB (deprived and skilled) from 162 countries.	Universal Health care regime, co-payments and private health services dependent on income.	Increasing welfare dependency.
			Neustadt: 43,699 residents, 26 % PMB, students, migrants and middle-class. Decreasing welfare dependency with early gentrification. Long history migration.
Portugal	Lisbon:	Mediterranean regime	Lumiar: 25,000 residents, 15 % migrants, high welfare dependency, high deprivation.
	largest city	High levels of austerity and cuts and restructuring of welfare.	Mouraria: 15,000 residents, migrants from 30 countries since the 1970s. Welfare dependency paired with gentrification.
	547,733 residents		
	housing half Portugal’s migrants from 100 countries	Residents pay fee for health service redeemable with proof of economic need. Bureaucratic barriers to access.	
Sweden	Uppsala: 4th largest city.	Democratic, universal regime	Gottsunda: 9,924 residents, 53 % PMB, high welfare dependency. Long history migration. Significant municipal investment addressing social problems.
	202,625 residents, PMB from 174 countries (deprived and skilled)	Impermeable to irregular migrants. Minimal austerity cuts.	Sävja: 5,330 residents, 39 % PMB, pockets of deprivation and affluence. Few municipal resources.
			Occasional social unrest.
UK	Birmingham:	Liberal regime	Lozells and East Handsworth: 31,074 residents, 44.9 % FB, 89.2 % EM, 5^th^ most deprived ward.
	2^nd^ largest city	Austerity cuts	Long history migration with recent increases and diversification.
	1,073,045 residents	Restructuring of welfare state	
	22.2 % FB, 46.9 % EM from 187 countries	Healthcare free except irregular migrants	Edgbaston: 24,426 residents, 29.2 % FB, 42.2 % EM, 34^th^ most deprived ward. More recent history migration.
		Increasing welfare chauvinism	

Abbreviations: *PMB* Person of Migrant Background, *FB* Foreign Born, *EM* Ethnic Minorities – definitions and terminology vary by country so data are not comparable

### Street-mapping

The first stage consists of street-mapping wherein researchers observe and record micro-level health-related activities underway in each superdiverse neighbourhood [35]. Undertaking online searches and participant observation in each superdiverse neighbourhood, the research teams will immerse themselves in and familiarise themselves with the localities. Structured ethnographic observation involving the capture of information about the local environment, local population activity and diversity, health related facilities and other faith based and community facilities will be conducted in each neighbourhood. In so doing – and through the annotation of maps, taking photographs of buildings housing health activities, talking to local people and the production of area pen portraits –will enable the identification of informal and private sector health services rarely appearing on official databases. The fieldwork will be carried out by academic research staff together with so-called community researchers. Community researchers are poly-lingual local residents who will receive post-graduate level research training [36] and are recruited via community organisations and local networks. The community researchers will utilise their language skills, local networks and knowledge to assist with the identification of services and in accessing respondents. This phase of the study will enable the development of connections with providers and residents for subsequent interviews. Overall, the street-mapping will contribute to a detailed understanding of the neighbourhoods and supporting a first comparison within and between the four countries.

#### Mobile phone application

The data collected from the street-mapping will be entered into a database providing the basis for a mobile phone application. The data will be inputted into the app by our researchers, residents and providers, all of who will be trained in its use. While smartphone usage is limited to 50 % of the general population, ownership rates are often higher within superdiverse and deprived areas because they are viewed as more cost effective than landlines/broadband and mobility is valued [37]. Allowing the identification of services and GIS mapping of utilisation, the app will identify location provision-clusters and allow residents to search and locate services. This application will eventually provide a crowd-sourced recommendation system enabling the on-going input and updating of information about healthcare services across all sectors via smart phone. While the primary aim of the app is to offer a resource for local communities to improve access to health services it will also aid us to build a resident-led picture of provision and, subject to its take-up, access to further funding, and additional ethical approval, we will explore the possibility of using the app to location track respondents in relation to their access to healthcare.

### In-depth interviews with residents and providers

In-depth interviews will detail how residents identify, utilise and combine services. Researchers and community researchers will undertake paired interviews with a maximum diversity sample [38] of 20 residents from each neighbourhood, using the resident’s preferred language. This purposive sampling suits diverse small sample sizes with limited population information. We will identify our sample through the street-mapping, guarding against over-reliance on community organisations and consequent under-representation of isolated or self-sufficient residents. Residents with different combinations of variables including majority, new and old residents, country of origin, migration status, income, education, age, gender, religion, and linguistic ability will be included. The interviews will explore the help-seeking experiences of all types of residents including the ways they experience, communicate, access and address health need within and beyond neighbourhood boundaries (i.e. national and transnational networks) - digitally and corporeally - and the factors that impact on experiences. Each interview will be recorded and translated into the local research team’s language.

Ten in-depth interviews with providers from public/private/third sectors identified by residents and street-mapping in each neighbourhood will be carried out. The interviews will focus on the systems in which professionals operate, including how they identify and respond to need, the impact of local and national regimes and the challenges faced.

Using ATLAS Ti, the research teams from the four countries will collaboratively analyse interview data developing an analytical framework constructed from codes created through team systematic thematic analysis of a 20 % sample of transcripts translated into English. Thematic analysis involves categorizing qualitative material – in this case interviews – according to its content and sense, to discern patterns of meaning recurring across interviews [39]. Each team will then code their own data and meet again to identify different models of welfare bricolage. This knowledge is then used to create the set of relevant questions for the survey.

### Health survey

In each superdiverse neighbourhood, we will undertake a resident survey testing the welfare bricolage models identified in the qualitative analysis to explore which type of models are adopted by which type of residents within or between different neighbourhoods and countries. The survey questions will be standardised in each country but translated into local languages. Cognitive testing will be carried out to check question wording and questionnaire length. Full piloting will be performed to test the questionnaire in the field and fieldwork procedures. The community researchers, with their local knowledge/languages, will be part of the survey team. Interviews will be conducted face to face.

#### Sampling and recruitment

The target group of this survey includes all adults living in one of the eight neighbourhoods regardless of their background. The participation of migrants or ethnic minorities in health surveys is typically lower than in the majority population [40, 41]. Research has indicated that a combination of register-based and community-based sampling approaches contributes to survey participation of diverse population groups [41]. Both approaches will be used in this survey. In the first step, a register-based approach will be applied including random sampling from the residents’ registration office. Participants will be contacted via the post and informed about the study. Information materials will be provided in several languages. As some of the residents in the neighbourhoods may be undocumented, it is necessary to use additional sampling strategies. Community-based sampling strategies will, for example, entail using the community networks established through the various research activities including street-mapping, in-depth interviews and distribution of study materials in the neighbourhoods.

There are no exact assumptions for the survey’s sample size determination as the research hypotheses (endpoints and determinants) will be generated via ethnographic investigation. We roughly aim for 4,800 responses, 600 in each neighbourhood, to allow for comparisons across neighbourhoods and countries.

#### Statistical analysis

Using multivariate analysis, we will systematically explore the relationships between welfare regimes and welfare bricolage models, and develop new knowledge about the types of models utilised by individuals with different characteristics across neighbourhood, national and transnational levels. Descriptive statistics will initially profile each neighbourhood. Multivariate analysis will be conducted to model welfare bricolage based on individual and context specific factors. Depending on the measurement of our dependent variables, possible statistical models include binary and ordinal logistic regressions, and structured equation models.

### Ethics statement and consent

Ethical approval for all study procedures was obtained from the Ethical Review Committee of the University Birmingham. All participants in the in-depth interviews and the health survey will receive written and/or oral information about the study. All interviewees will give informed consent for their data to be used.

## Discussion

The findings of this study will help practitioners and policymakers understand changes in health needs, in order to shape services for residents living in superdiverse areas. It will provide academics with a new framework to utilise when researching health at local level. Bringing the concept of bricolage together with superdiversity has high potential for scientific impact because it provides a concept with sufficient flexibility and creativity to capture practices evolving in response to increased complexity, interactions and transnational connections. In addition, methodological developments which incorporate thick data collection via ethnographic approaches, inductive theory development through interdisciplinary reflection and subsequent use of epidemiological surveys to verify ideas, is highly innovative and will bring new skills and knowledge.

Using an interdisciplinary and collaborative approach, the UPWEB study will bring insights to public health, social policy and migration studies, and will provide both concept and method development enabling new knowledge to be gathered about the ways in which welfare services are understood, interacted with and combined to meet the needs of highly complex superdiverse and frequently hypermobile populations. By introducing welfare bricolage, the study provides a model for combining methods, disciplines and sectors. By comparing neighbourhoods and countries we identify universal and exceptional practice and develop an understanding of the influence of national and local welfare states on practice. By utilising a community researcher approach and training academics to train community researchers we are building future capacity at the local level to reutilise these techniques.

A limitation of this study is that it includes only eight neighbourhoods in four countries. A larger number of neighbourhoods and countries would be desirable in order to draw generalisations regarding welfare state regimes. With only one country from each regime type in our sample, we will not be able to assess within-regime type variability. Hence, generalisation of welfare bricolage practices to welfare state regimes will only be possible on theoretical grounds. Furthermore, one may be critical about the complexity and fluidity in the notions of superdiversity and bricolage which seem to interfere with scientific goal of drawing generalisation from local findings (and, thereby, reduce complexity). Although both superdiversity and bricolage aim to provide a more nuanced understanding, they are not anti-categorical concepts. Rather, the concepts imply the usage of more fine-grained categories and typologies. In this context the application of qualitative research methods plays an important role, because relevant categories are not predefined but developed and refined during the analysis of the empirical material. Grounding the categories and their interplay in the empirical material and comparing them across the neighbourhoods and countries will be the strategy in this study to move beyond purely local knowledge production.

The use of community researchers may also be subject of criticism. Community researchers can offer valuable insights into the local structures in the neighbourhoods, but they may also introduce bias. For example, there may be a lack of neutrality in community researchers’ perception of the neighbourhood or they may inadvertently direct the focus of the research towards their own ethnic community. To avoid these kinds of bias, street-mapping and in-depth interviews are carried out by pairs of community and academic researchers.

As for all epidemiological surveys, the inclusion of marginalised groups, such as persons with no or restricted residence, is a major challenge for the health survey. Although a random sample provides desirable properties for statistical analyses, standard sampling procedures have shown limited success in migrant health research [40, 41]. Hence, a variety of recruitment strategies will be applied to reach the different groups in the neighbourhoods. The recruitment does primarily not aim for inclusion of the samples of ethnic, religious or differently defined groups according to their population-based proportion in the neighbourhood, but to boost the sample to ensure that we incorporate the dimensions of population complexity evident in each neighbourhood enabling us to reach sufficient numbers for comparison. Nevertheless, given the extent of difference, along wide-ranging lines (i.e. ethnicity, country of origin, legal status etc.), it will not be possible to include a representative sample of all groups.

In spite of these limitations we expect that findings from this study covering the range of practices utilised and the role of providers in complex environments will help shape future services, providing knowledge about how welfare can be reconstructed to address need associated with complex life worlds. It will support the development of new networks and partnerships that can collaborate to meet need at local level. The concept of welfare bricolage will offer practitioners a new mechanism to begin to understand the wide range of resources that residents harness to attempt to meet individual need. With this information they can make their practices more appropriate and collaborate with other providers, and residents, to make resources available or design new responses.

## Abbreviations

EM: Ethnic minority
FB: Foreign-born
GIS: Geographical information system
PMB: Person of migrant background
UPWEB: Understanding the practice and developing the concept of welfare bricolage
